# ABO Blood Type Associations with Physical, Mental, and Biochemical Characteristics in a Taiwanese Population

**DOI:** 10.3390/life15121793

**Published:** 2025-11-21

**Authors:** Yu-Ting Yan, Eugene Lin, Mu-N Liu, Po-Hsiu Kuo, Ding-Lieh Liao, Shih-Jen Tsai

**Affiliations:** 1 Department of Public Health, Institute of Epidemiology and Preventive Medicine, National Taiwan University, Taipei 100, Taiwan; yutingyan0101@gmail.com (Y.-T.Y.); phkuo@ntu.edu.tw (P.-H.K.); 2 Pacific Northwest Research Institute, Seattle, WA 98122, USA; lines@uw.edu; 3 Department of Biostatistics, University of Washington, Seattle, WA 98195, USA; 4 Graduate Institute of Biomedical Sciences, China Medical University, Taichung 404, Taiwan; 5 Department of Psychiatry, Taipei Veterans General Hospital, Taipei 112, Taiwan; liumuen@gmail.com; 6 Division of Psychiatry, School of Medicine, College of Medicine, National Yang Ming Chiao Tung University, Taipei 112, Taiwan; 7 Department of Psychiatry, National Taiwan University Hospital, Taipei 100, Taiwan; 8 Department of General Psychiatry, Bali Psychiatric Center, Ministry of Health and Welfare, New Taipei City 249, Taiwan; dlliao777@gmail.com

**Keywords:** ABO, Taiwan Biobank, physical disease, mental disease, biochemical variables

## Abstract

The ABO blood group system, characterized by specific glycosyltransferase activity, is linked to various biological and clinical traits. However, findings often lack consistency due to small sample size and ethnic variability. This research explores potential associations between ABO blood types and physical, mental, and physiological traits in a substantial Taiwanese population. We utilized data from 146,374 individuals in the Taiwan Biobank. Compared to people with blood type O, those with blood types A and B exhibited a reduced risk of peptic ulcers (11% and 8%, respectively). Blood type A was associated with a 16% increased risk of hyperlipidemia and an 18% increased risk of asthma. It also had 2.3% higher total cholesterol and 3.3% higher LDL-C than type O. Blood type B showed 1.6% higher triglyceride levels. This study observes possible associations between ABO blood types and physical diseases, including gastric ulcers and asthma, as well as unique biochemical profiles. The results suggest that ABO blood typing may offer supplementary value in population-level risk assessment. Future research should elucidate underlying mechanisms and validate findings across populations.

## 1. Introduction

The ABO blood group system, discovered by Karl Landsteiner in 1901, categorizes individuals based on the presence of antigens A and B on red blood cells (RBCs) [1]. The system is genetically determined by the ABO gene located on chromosome 9, and its alleles encode glycosyltransferase enzymes responsible for these antigens. Beyond transfusion compatibility, these antigens play a crucial role in immunological processes and have been linked to various physical diseases.

There exists a well-documented correlation between ABO blood types and physical ailments, indicating that individuals with non-O blood groups (A, B, and AB) exhibit an elevated risk of developing cardiovascular diseases, such as coronary artery disease (CAD), myocardial infarction, and venous thromboembolism (VTE) [2, 3]. A meta-analysis of 10 trials with 174,945 participants was performed to assess the correlation between non-O blood types and the risk of coronary artery disease (CAD) [3]. The analysis revealed that individuals with non-O blood groups have a 14% higher risk of CAD compared to those with O blood groups. Furthermore, non-O blood groups were associated with a 16% higher risk of AMI [3]. In a study using data from 406,755 individuals in the UK Biobank, Groot et al. found blood group B had a 1.15 times increased risk of myocardial infarction. Conversely, non-O blood groups showed a lower risk of hypertension compared to blood group O [4]. This increased risk is mainly due to enhanced levels of von Willebrand factor and factor VIII in individuals with non-O blood types [5, 6]. Besides cardiovascular problems, research has indicated possible associations between ABO blood type and autoimmune diseases [7], metabolic disorders including type 2 diabetes [8], several malignancies, including gastric, pancreatic, and colorectal cancers [9], as well as numerous infections [10, 11].

Studies on the relationship between ABO blood types and mental diseases have produced conflicting results. Some research suggests that AB blood type is more common in mental disorder patients [12], and there may be a link between AB type and increased anxiety [13]. Other investigations indicated a relationship between blood type O and bipolar disorder or major depressive disorder (MDD), while type A was connected to involutional depression [14]. However, a large-scale pooled analysis with data from 26 cohorts revealed no significant link between ABO blood types and MDD [15]. Certain studies discovered that type A persons had greater obsessive–compulsive disorder than type O individuals [16].

The relationship between ABO blood types and physical or mental diseases has been the subject of many studies, but findings often show inconsistencies. These disparities can be ascribed to several factors. Studies conducted across diverse populations may produce disparate outcomes owing to variations in genetic variety. The geographical distribution of ABO blood types varies, affecting outcomes in particular groups [17]. Moreover, limited or non-representative sample sizes in research may result in statistical abnormalities or findings that lack generalizability.

Limited research has investigated the correlation between ABO blood types and physical or psychological disorders in the Asian population. This study has two objectives: first, to ascertain the relationship between ABO blood types and physical or mental diseases, and second, to investigate the correlation of these ABO blood type with various biochemical parameters in a substantial Taiwanese cohort.

## 2. Materials and Methods

### 2.1. Subjects

The study cohort included 146,239 Taiwanese participants from the Taiwan Biobank [18, 19, 20, 21]. From 2013 to 2022, the Taiwan Biobank enlisted participants from the diverse Taiwanese population across Taiwan, gathering specimens and pertinent data via recruitment sites [19, 20]. The Taiwan Biobank, predominantly funded by the Taiwanese government, seeks to advance scientific study on various public health concerns and foster collaboration among researchers addressing prevalent local chronic diseases [21]. Individuals who self-identified as Taiwanese descendants, were a minimum of twenty years old, and could independently do daily tasks were eligible for recruitment [18, 22].

Before commencing the study, the Institutional Review Board of Taipei Veterans General Hospital granted ethical approval (approval number: 2023-04-007CC#1), and all participants provided informed consent in accordance with established regulations and laws.

### 2.2. Genotyping

DNA extraction from blood samples was performed utilizing the QIAamp DNA blood kit following the manufacturer’s instructions (Qiagen, Valencia, CA, USA). SNP genotyping was conducted with the Axiom Genome-Wide Array Plate System (Affymetrix, Santa Clara, CA, USA) with custom Taiwan Biobank chips. Genetic determination of ABO blood types involves analyzing the ABO gene on chromosome 9q34.2, which encodes glycosyltransferases responsible for synthesizing A and B antigens. Two primary single nucleotide polymorphism (SNP) combinations were used to define ABO blood types in the study [23, 24]:

Combination 1 (rs8176719 and rs8176746): rs8176719 is linked to the O blood type, while rs8176746 is associated with the B blood type (Supplemental Table S1).

Combination 2 (rs8176719, rs635634 and rs7030248): rs635634 is connected to the A blood type, whereas rs7030248 is associated with the B blood type (Supplemental Table S2).

For blood type classification, we remove individuals whose blood types are inconsistent with the two methodologies or cannot be ascertained by either procedure, as illustrated in Table 1.

### 2.3. Disease Diagnoses

Disease diagnoses are based on self-reporting. The questionnaire includes inquiries such as, “Have you received a diagnosis of asthma from a physician?” The age at diagnosis is not included in the questionnaire, and medication use, or hospital-confirmed records were not available for most traits in the current dataset.

### 2.4. Statistics

We utilized logistic regression models to analyze the association between clinical diseases and blood types, controlling for age, sex, and the first 10 principal components. The correlation between biochemical variables and blood types was analyzed by linear regression models, adjusted for age, sex, and the first 10 principal components. The threshold for significance was established at *p* < 0.05. The Bonferroni correction was employed to control for multiple testing. Data are expressed as the mean ± standard deviation (SD).

## 3. Results

A total of 146,374 individuals—53,006 males and 93,368 women—aged between 20 and 90 years (mean: 49.42, SD: 11.38 years) were examined. Table 1 presents the results of the Combination 1 and 2 for ascertaining ABO blood types. The distributions exhibit considerable similarity between both methods, indicating that either methodology yields consistent blood type classification. Table 2 presents the baseline characteristics categorized by ABO blood type.

Supplemental Table S3 illustrates the prevalence of diverse physical and mental diseases among a population of 144,550 individuals. Chronic illnesses such as peptic ulcer (14.23%, 20,474 cases), gastroesophageal reflux (14.14%, 20,348 cases), and hypertension (12.14%, 17,418 cases) were frequent. For mental diseases, depression (3.67%, 5276 cases) was the most frequently reported condition.

Table 3a,b and Supplemental Table S3 present the odds ratios (OR) and *p*-values for associations between different ABO blood types (A, B, AB) and type O (reference group), and their relationships with various physical and mental diseases. For physical illnesses, significant associations were observed between ABO blood types and several conditions. Blood types A and B were associated with a significantly lower risk of peptic ulcers compared to blood type O (A vs. O: OR = 0.89, *p* < 0.0001; B vs. O: OR = 0.92, *p* < 0.0001). Compared to blood type O, blood type A was linked to a slightly reduced risk of hypertension. However, individuals with blood type A showed a higher risk of hyperlipidemia compared to other groups, suggesting a notable relationship between blood type A and lipid metabolism. Furthermore, blood type A was associated with a significantly increased risk of asthma compared to blood type O (A vs. O: OR = 1.18, *p* < 0.0001).

For mental diseases, blood type AB demonstrated a potential association with a higher risk of depression and obsessive–compulsive disorder, though neither met the corrected significance threshold. These findings suggest potential links between ABO blood types and susceptibility to certain psychiatric conditions.

There are notable variations among ABO blood types, according to the biochemical characteristics analyzed (Table 4a,b and Supplemental Table S4). Blood type A was associated with significantly lower platelet counts and hematocrit levels compared to blood type O, alongside higher levels of total cholesterol and LDL-C. Similar patterns were observed for blood type AB, which demonstrated reduced WBC counts, lower platelets, and elevated LDL-C. Blood type B also showed significant reductions in platelet counts but was linked to higher triglyceride levels.

Comparisons between non-O blood groups further highlighted distinctions. Blood type A had significantly higher levels of total cholesterol and LDL-C compared to blood type B. Hematocrit levels were also significantly lower in type A relative to type B and AB.

## 4. Discussion

This study provides a comprehensive evaluation of the association between ABO blood types and various physical and mental diseases, as well as their correlation with biochemical markers, within a large Taiwanese population. The findings reveal notable differences in disease susceptibility and biochemical profiles among ABO blood groups.

Our study identified significant associations between ABO blood types and various physical illnesses (Table 3a,b). Notably, individuals with non-O blood types exhibited a reduced risk of peptic ulcers compared to type O, with consistent statistical significance. In 1956, Buckwalter et al. first demonstrated that type O was more frequent among the ulcer patients (n = 1301) than in the reference group (n = 8767) [25]. This association has been observed by later studies across different populations, including Swedish, Danish, Japanese, and Ethiopian cohorts [26, 27, 28]. However, some research challenged this, suggesting equal risk for blood types O and A [29]. Current study is the first report to our knowledge which indicates that the risk of peptic ulcers is lower in Chinese individuals with non-O blood types than in those with type O. The increased prevalence of peptic ulcers among individuals with blood type O has been substantiated by various studies, which have explored several potential reasons for this association: First, individuals with blood type O may have an increased susceptibility to Helicobacter pylori infection, a significant risk factor for peptic ulcer disease. A study found that Helicobacter pylori binds more effectively to the epithelial cells of individuals with blood group O, leading to higher inflammatory responses [30]. Second, individuals with blood type O have been observed to exhibit higher levels of pro-inflammatory cytokines in response to Helicobacter pylori infection [30]. This heightened inflammatory response can lead to increased gastric mucosal damage, thereby elevating the risk of peptic ulcer development.

Conversely, blood type A was associated with increased risks of hyperlipidemia. Blood type A was associated with higher total cholesterol levels and an increased risk of dyslipidemia in French women [31]. Blood group A was associated with elevated total cholesterol and low-density lipoprotein cholesterol (LDL-C) values across diverse populations [32, 33, 34]. The UK Biobank study indicated that blood group A was correlated with elevated levels of many lipoprotein characteristics, notably LDL-C, high-density lipoprotein cholesterol (HDL-C), total cholesterol, apolipoprotein A, and apolipoprotein B [35]. Similarly, our study identified that blood groups A and AB were associated with elevated total cholesterol and LDL-C levels in comparison to blood types B and O (Table 4a,b). Genome-wide association studies have identified ABO gene variants as major determinants of soluble E-selectin (sE-selectin) levels, with pleiotropic effects on total and LDL-C [36]. Research suggests a significant association between sE-selectin levels and blood lipids. Elevated sE-selectin levels are observed in patients with hypertriglyceridemia and low HDL-C [37]. Positive correlations between sE-selectin and triglycerides, LDL-C, and negative correlations with HDL-C have been reported in atherosclerosis patients [38]. Further investigation is required to determine whether the correlation between ABO blood types and blood lipids is mediated by sE-selectin.

This study found that blood type A is linked to an 18% higher risk of asthma compared to blood group O (Table 3a,b). Research on the relationship between blood types and asthma risk has yielded mixed results. Some studies suggest that blood group A may be associated with an increased risk of asthma [39, 40], while others indicate that blood group O may be linked to a higher susceptibility to asthma exacerbations [41]. Individuals with blood type A showed a higher risk of asthma, suggesting a role of ABO antigens in airway inflammation and immune regulation. Because ABO typing is simple and inexpensive, it may serve as a supplementary marker for early identification of individuals with heightened susceptibility to asthma.

The relationship between ABO blood types and CAD has been the subject of numerous investigations. In this investigation, ABO blood types were not linked to CAD risk (Supplemental Table S3). In one study conducted in Bangladesh, blood group O was related with a higher incidence of CAD [42]. Non-O blood group, on the other hand, has been linked to an increased risk of CAD in some studies [43, 44]. The association between ABO blood group and CAD is still debated. In 2016, Chen et al. conducted a meta-analysis of 17 related studies and discovered that the risk of CAD was slightly higher in blood group A than in blood group O [45].

Our investigation showed no link between ABO blood types and MDD or OCD (Supplemental Table S3a,b). Research on the association between ABO blood types and MDD has yielded mixed results [15]. Although the results point to potential associations between ABO blood types and mental illnesses, in many situations their interpretation is constrained by marginal statistical significance.

The cross-population comparison between the Taiwan Biobank and UK Biobank highlights both convergent and divergent biological patterns linking genetic background, disease susceptibility, and metabolic traits (Table 5). Consistent findings were observed for cardiovascular and vascular indicators—non-O blood types in Taiwan showed slightly lower hypertension risk, paralleling the inverse genetic correlations between educational attainment and blood pressure or coronary artery disease in the UK sample. However, metabolic traits displayed opposite directions: blood type A individuals exhibited higher LDL-C, total cholesterol, and HbA1c in Taiwan, whereas higher cognitive or educational scores in the UK Biobank correlated with lower cardiometabolic genetic risk. Collectively, these findings suggest that while vascular pathways may share conserved pleiotropic mechanisms across ethnicities, lipid and glucose metabolism could reflect population-specific genetic or environmental influences that warrant deeper functional validation.

The analysis of biochemical markers indicated considerable differences between ABO blood types (Table 4a,b). The results revealed that individuals with blood type A exhibited lower hematocrit and platelet counts, while those with blood type B showed higher red blood cell counts compared to type O. These findings expand upon prior studies predominantly centered on cardiovascular and metabolic characteristics or on vWF/FVIII. The association of blood type A with lower platelet counts and hematocrit levels, alongside elevated total and LDL-C, raises concerns about its potential link to cardiovascular risk. Increased cholesterol levels are well-known risk factors for atherosclerosis, while lower hematocrit and platelet counts could impact coagulation and oxygen transport [46, 47]. Similarly, the lipid profile observed in individuals with blood type AB, including elevated LDL-C, reinforces this concern, compounded by their lower WBC counts, which may reflect an altered immune response.

The main advantage of this study is the substantial sample size derived from the Taiwan Biobank, facilitating the examination of correlations between ABO blood type and physical, mental, and biochemical characteristics among the four blood types in a Taiwanese cohort. However, several limitations should be acknowledged. First, this study is based on a Taiwanese population, and the findings may not be generalizable to other ethnic or geographic groups. Second, while the study accounted for the biochemical parameters and disease prevalence, additional factors such as environmental exposures and genetic interactions were not assessed. Third, the reliance on cross-sectional data limits causal inferences. Finally, medical diagnoses relied on self-reported data, which are intrinsically susceptible to recall bias, misclassification, and social desirability bias [48].

Limitations: This study is based on cross-sectional data, which limits our ability to make causal inferences. Additionally, disease outcomes were based on self-reported data, increasing the risk of misclassification. Moreover, although many associations were statistically significant, we acknowledge that several effect sizes are modest. Nonetheless, we believe these findings may still be relevant for population-level risk stratification and predictive modeling in precision medicine. The early detection of those who are more susceptible to metabolic and inflammatory illnesses may be improved by including ABO typing into predictive algorithms.

## 5. Conclusions

This study highlights significant associations between ABO blood types and physical illnesses, as well as their correlation with key biochemical markers. These results provide new findings into the biological underpinnings of disease susceptibility and pave the way for future research aimed at leveraging ABO blood type information for precision medicine. Although many associations were statistically significant due to the large sample size, effect sizes were modest. Still, the early detection of those who are more susceptible to metabolic and inflammatory illnesses may be improved by including ABO typing into predictive algorithms.

## Figures and Tables

**Table 1:** The distribution of ABO blood types as defined by two single nucleotide polymorphism (SNP) combination methodologies.

		Defined by rs8176746 and rs8176719					
		A	AB	B	O	<NA>	Total
Defined by rs8176719, rs635634 and rs7030248	A	37,338	16	296	2	0	37,652
	AB	251	8468	40	0	0	8759
	B	905	221	35,257	0	42	36,425
	O	0	0	0	62,708	0	62,708
	<NA>	284	80	173	200	93	830
	Total	38,778	8785	35,766	62,910	135	146,374

ABO blood types are included in the analysis if determined by at least one of the two SNP groups. If the blood type cannot be determined from one SNP group (resulting in NA), it is still included if determined by the other group. However, cases where the blood type is inconsistent between the two methods are excluded. In the end, 37,622 individuals were classified as type A, 8548 as type AB, 35,472 as type B, and 62,908 as type O.

**Table 2:** Baseline characteristics stratified by ABO blood type.

	A (N = 37,622)	AB (N = 8548)	B (N = 35,472)	O (N = 62,908)	*p*-Value
AGE-Mean (SD)	49.39 (11.37)	49.67 (11.31)	49.45 (11.38)	49.38 (11.38)	0.148
BMI-Mean (SD)	24.29 (3.86)	24.19 (3.87)	24.28 (3.88)	24.27 (3.82)	0.247
Gender-n (%)					
Male	13,669 (36.3)	3108 (36.4)	12,882 (36.3)	22,702 (36.1)	0.826
Femal	23,953 (63.7)	5440 (63.6)	22,590 (63.7)	40,206 (63.9)	
Smoking-n (%)					
Ever smoked for more than 6 months	7356 (19.6)	1774 (20.8)	7079 (20.0)	12,375 (19.7)	0.058
Never smoked for ≥6 months	30,252 (80.4)	6771 (79.2)	28,380 (80.0)	50,509 (80.3)	
Acohol-n (%)					
No or occasional drinking,	34,291 (91.2)	7810 (91.4)	32,305 (91.1)	57,391 (91.3)	0.127
Former drinker	941 (2.5)	237 (2.8)	949 (2.7)	1712 (2.7)	
Current drinker	2355 (6.3)	497 (5.8)	2195 (6.2)	3754 (6.0)	

Continuous variables are shown as mean ± SD and compared using ANOVA. Categorical variables are shown as n (%) and compared using chi-square test.

**Table 3:** (**a**): Significant associations between blood type and physical diseases (O as reference). (**b**): Significant associations between blood type and physical diseases (pairwise comparisons).

**(a)**						
	**A vs. O**		**AB vs. O**		**B vs. O**	
	**OR (95% CI)**	***p*-Value (BH-FDR)**	**OR (95% CI)**	***p*-Value (BH-FDR)**	**OR (95% CI)**	***p*-Value (BH-FDR)**
Valve heart disease	1.08 (1.01–1.15)	0.0253 (0.1096)	1.09 (0.97–1.21)	0.1393 (0.5174)	1.02 (0.96–1.09)	0.5442 (0.8268)
Hyperlipidemia	1.16 (1.1–1.22)	<0.0001 * (<0.0001)	1.04 (0.96–1.14)	0.3399 (0.6312)	1.03 (0.98–1.09)	0.2088 (0.8190)
Hypertension	0.94 (0.9–0.98)	0.0055 (0.0357)	0.95 (0.89–1.03)	0.2176 (0.5543)	0.98 (0.94–1.02)	0.3325 (0.8190)
Depression	0.99 (0.93–1.06)	0.8183 (0.9567)	1.12 (1.00–1.26)	0.0470 (0.2662)	0.99 (0.93–1.07)	0.8536 (0.9631)
Obsessive–compulsive disorder	0.97 (0.65–1.45)	0.8870 (0.9567)	1.84 (1.08–3.15)	0.0256 (0.2219)	1.11 (0.75–1.64)	0.5950 (0.8268)
Epilepsy	0.95 (0.77–1.18)	0.6575 (0.8997)	1.49 (1.08–2.04)	0.0137 (0.1781)	0.98 (0.79–1.22)	0.8826 (0.9631)
Parkinson’s disease	0.55 (0.36–0.85)	0.0069 (0.0359)	1.17 (0.65–2.10)	0.6037 (0.8107)	0.98 (0.69–1.41)	0.9261 (0.9631)
Peptic ulcer disease	0.89 (0.86–0.92)	<0.0001 * (<0.0001)	0.89 (0.83–0.95)	0.0005 * (0.0130)	0.92 (0.88–0.95)	<0.0001 * (<0.0001)
Asthma	1.18 (1.11–1.26)	<0.0001 * (<0.0001)	1.02 (0.90–1.15)	0.7894 (0.8971)	1.05 (0.98–1.12)	0.1881 (0.8190)
Liver gall stone	1.00 (0.94–1.06)	0.9567 (0.9567)	1.05 (0.94–1.17)	0.3662 (0.6347)	0.94 (0.88–1.00)	0.0520 (0.6760)
**(b)**						
	**A vs. B**		**A vs. AB**		**B vs. AB**	
	**OR (95% CI)**	***p*-Value (BH-FDR)**	**OR (95% CI)**	***p*-Value (BH-FDR)**	**OR (95% CI)**	***p*-Value (BH-FDR)**
Valve heart disease	1.05 (0.98–1.13)	0.1549 (0.3356)	0.99 (0.88–1.11)	0.8513 (0.9222)	0.94 (0.84–1.05)	0.2842 (0.7389)
Hyperlipidemia	1.12 (1.06–1.19)	0.0001 * (0.0026)	1.11 (1.01–1.22)	0.0239 (0.1461)	0.99 (0.90–1.09)	0.8313 (0.9727)
Hypertension	0.96 (0.92–1.01)	0.1130 (0.3006)	0.99 (0.91–1.07)	0.7405 (0.8648)	1.03 (0.95–1.11)	0.5171 (0.8403)
Depression	1.00 (0.92–1.08)	0.9705 (0.9705)	0.88 (0.78–1.00)	0.0417 (0.1807)	0.88 (0.78–1.00)	0.0453 (0.2752)
Obsessive–compulsive disorder	0.87 (0.57–1.35)	0.5461 (0.7473)	0.53 (0.30–0.93)	0.0281 (0.1461)	0.60 (0.34–1.06)	0.0793 (0.2945)
Epilepsy	0.97 (0.76–1.24)	0.7945 (0.8607)	0.64 (0.46–0.89)	0.0090 (0.1461)	0.66 (0.47–0.92)	0.0157 (0.2752)
Parkinson’s disease	0.56 (0.35–0.90)	0.0166 (0.1439)	0.47 (0.24–0.91)	0.0258 (0.1461)	0.84 (0.45–1.56)	0.5841 (0.8933)
Peptic ulcer disease	0.97 (0.93–1.01)	0.1409 (0.3330)	1.00 (0.93–1.07)	0.9951 (0.9951)	1.03 (0.96–1.11)	0.3705 (0.8004)
Asthma	1.13 (1.05–1.22)	0.0018 * (0.0234)	1.16 (1.03–1.32)	0.0184 (0.1461)	1.03 (0.91–1.17)	0.6387 (0.9226)
Liver gall stone	1.06 (0.99–1.14)	0.0880 (0.3006)	0.95 (0.85–1.06)	0.3684 (0.6779)	0.89 (0.80–1.00)	0.0485 (0.2752)

Using logistic regression models to investigate the association between clinical conditions and blood types, adjusted for age, sex, and the first 10 principal components (PCs). Only significant results are shown here, the full table is presented in Supplementary Table S3. *p*-values are shown with BH-FDR adjusted values in parentheses (BH-FDR was calculated across 26 tested diseases). Values with *p* < 0.05 are highlighted in orange, and those significant after Bonferroni correction are indicated with *.

**Table 4:** (**a**): Significant associations between blood types and biochemical variables (O as reference). (**b**): Significant associations between blood types and biochemical variables (pairwise comparisons).

**(a)**						
	**A vs. O**		**AB vs. O**		**B vs. O**	
	**BETA (95% CI per SD)**	***p*-Value (BH-FDR)**	**BETA (95% CI per SD)**	***p*-Value (BH-FDR)**	**BETA (95% CI per SD)**	***p*-Value (BH-FDR)**
SBP, mmHg	−0.002 (−0.013, 0.010)	0.7920 (0.8316)	−0.027 (−0.047, −0.007)	0.0087 (0.0255)	−0.013 (−0.025, −0.002)	0.0245 (0.0572)
DBP, mmHg	−0.013 (−0.025, −0.001)	0.0381 (0.0500)	−0.007 (−0.028, 0.0150)	0.5449 (0.6023)	−0.001 (−0.014, 0.011)	0.8231 (0.8380)
BMI	−0.002 (−0.015, 0.010)	0.7032 (0.7772)	−0.028 (−0.050, −0.006)	0.0138 (0.029)	−0.005 (−0.017, 0.008)	0.4785 (0.6699)
RBC, million/μL	−0.049 (−0.060, −0.038)	<0.0001 * (<0.0001)	−0.002 (−0.021, 0.018)	0.8631 (0.8631)	0.085 (0.073, 0.096)	<0.0001 * (<0.0001)
WBC, 1000/μL	−0.045 (−0.057, −0.032)	<0.0001 * (<0.0001)	−0.043 (−0.065, −0.020)	0.0002 * (0.0014)	−0.013 (−0.026, 0.000)	0.0521 (0.0995)
Platelet, 1000/μL	−0.029 (−0.041, −0.016)	<0.0001 * (<0.0001)	−0.027 (−0.048, −0.005)	0.0154 (0.0294)	−0.042 (−0.054, −0.029)	<0.0001 * (<0.0001)
Hematocrit, %	−0.048 (−0.059, −0.038)	<0.0001 * (<0.0001)	−0.019 (−0.037, −0.001)	0.0431 (0.0754)	0.047 (0.037, 0.057)	<0.0001 * (<0.0001)
HbA1c, %	0.025 (0.013, 0.038)	<0.0001 * (<0.0001)	0.027 (0.006, 0.048)	0.0126 (0.029)	0.002 (−0.010, 0.014)	0.7587 (0.8380)
Fasting glucose, mg/dL	0.026 (0.014, 0.038)	<0.0001 * (<0.0001)	0.012 (−0.009, 0.034)	0.2653 (0.3979)	0.011 (−0.002, 0.023)	0.0891 (0.1559)
Total cholesterol, mg/dL	0.132 (0.119, 0.144)	<0.0001 * (<0.0001)	0.046 (0.024, 0.069)	<0.0001 * (<0.0001)	0.013 (0.000, 0.026)	0.0512 (0.0995)
Triglyceride, mg/dL	−0.001 (−0.013, 0.012)	0.9119 (0.9119)	0.013 (−0.009, 0.035)	0.2383 (0.3849)	0.026 (0.013, 0.039)	0.0001 * (0.0004)
LDL-C, mg/dL	0.127 (0.114, 0.140)	<0.0001 * (<0.0001)	0.041 (0.019, 0.064)	0.0003 * (0.0016)	0.006 (−0.007, 0.019)	0.3390 (0.5085)
Albumin, g/dL	−0.018 (−0.031, −0.006)	0.0036 (0.0054)	0.012 (−0.010, 0.033)	0.2953 (0.4134)	0.057 (0.044, 0.069)	<0.0001 * (<0.0001)
AST, U/L	−0.032 (−0.045, −0.020)	<0.0001 * (<0.0001)	−0.029 (−0.051, −0.007)	0.0097 (0.0255)	0.003 (−0.010, 0.016)	0.6384 (0.7548)
ALT, U/L	0.024 (0.012, 0.036)	0.0001 * (0.0002)	0.035 (0.013, 0.057)	0.0017 * (0.0059)	0.040 (0.028, 0.053)	<0.0001 * (<0.0001)
Gamma-GT, U/L	0.032 (0.020, 0.044)	<0.0001 * (<0.0001)	0.041 (0.020, 0.063)	0.0002 * (0.0014)	0.020 (0.008, 0.033)	0.0016 * (0.0042)
BUN, mg/dL	0.018 (0.006, 0.029)	0.0033 (0.0053)	0.006 (−0.014, 0.027)	0.5418 (0.6023)	−0.020 (−0.031, −0.008)	0.0014 * (0.0042)
Creatinine, mg/dL	−0.019 (−0.028, −0.010)	<0.0001 * (<0.0001)	−0.028 (−0.044, −0.013)	0.0004 * (0.0017)	−0.003 (−0.012, 0.006)	0.5367 (0.7044)
Uric acid, mg/dL	−0.013 (−0.024, −0.002)	0.0202 (0.0283)	−0.008 (−0.027, 0.011)	0.4278 (0.5285)	−0.001 (−0.012, 0.010)	0.8380 (0.8380)
**(b)**						
	**A vs. B**		**A vs. AB**		**B vs. AB**	
	**BETA (95% CI per SD)**	***p*-Value (BH-FDR)**	**BETA (95% CI per SD)**	***p*-Value (BH-FDR)**	**BETA (95% CI per SD)**	***p*-Value (BH-FDR)**
SBP, mmHg	0.012 (−0.001, 0.025)	0.0736 (0.0966)	0.025 (0.005, 0.046)	0.0172 (0.0602)	0.014 (−0.007, 0.035)	0.2039 (0.2855)
DBP, mmHg	−0.011 (−0.025, 0.002)	0.1036 (0.1156)	−0.006 (−0.028, 0.016)	0.5848 (0.8103)	0.005 (−0.017, 0.027)	0.6481 (0.7224)
BMI	0.002 (−0.012, 0.016)	0.7637 (0.7637)	0.025 (0.002, 0.048)	0.0304 (0.0912)	0.023 (0.000, 0.046)	0.0490 (0.0935)
RBC, million/μL	−0.134 (−0.146, −0.121)	<0.0001 * (<0.0001)	−0.048 (−0.068, −0.027)	<0.0001 * (<0.0001)	0.086 (0.066, 0.107)	<0.0001 * (<0.0001)
WBC, 1000/μL	−0.032 (−0.046, −0.018)	<0.0001 * (<0.0001)	−0.002 (−0.025, 0.021)	0.8583 (0.8845)	0.030 (0.006,0.053)	0.0124 (0.0325)
Platelet, 1000/μL	0.013 (−0.001, 0.027)	0.0666 (0.0932)	−0.002 (−0.024, 0.021)	0.8678 (0.8845)	−0.015 (−0.037, 0.008)	0.1962 (0.2855)
Hematocrit, %	−0.096 (−0.107, −0.084)	<0.0001 * (<0.0001)	−0.030 (−0.049, −0.011)	0.0017 * (0.0089)	0.066 (0.047, 0.084)	<0.0001 * (<0.0001)
HbA1c, %	0.024 (0.010, 0.037)	0.0007 * (0.0015)	−0.002 (−0.024, 0.021)	0.8845 (0.8845)	−0.025 (−0.047, −0.003)	0.0265 (0.0556)
Fasting glucose, mg/dL	0.015 (0.001, 0.029)	0.0350 (0.0565)	0.014 (−0.009, 0.036)	0.2405 (0.5576)	−0.001 (−0.024, 0.021)	0.8980 (0.898)
Total cholesterol, mg/dL	0.119 (0.105, 0.133)	<0.0001 * (<0.0001)	0.085 (0.062, 0.109)	<0.0001 * (<0.0001)	−0.034 (−0.057, −0.010)	0.0046 (0.0161)
Triglyceride, mg/dL	−0.027 (−0.041, −0.012)	0.0002 * (0.0005)	−0.014 (−0.037, 0.009)	0.2318 (0.5576)	0.013 (−0.010, 0.036)	0.2828 (0.3712)
LDL-C, mg/dL	0.121 (0.106, 0.135)	<0.0001 * (<0.0001)	0.086 (0.063, 0.109)	<0.0001 * (<0.0001)	−0.035 (−0.058, −0.011)	0.0037 (0.0155)
Albumin, g/dL	−0.075 (−0.089, −0.061)	<0.0001 * (<0.0001)	−0.030 (−0.052, −0.007)	0.0095 (0.0399)	0.045 (0.023, 0.068)	0.0001 * (0.0007)
AST, U/L	−0.035 (−0.049, −0.021)	<0.0001 * (<0.0001)	−0.003 (−0.026, 0.019)	0.7648 (0.8845)	0.032 (0.009, 0.055)	0.0062 (0.0186)
ALT, U/L	−0.016 (−0.030, −0.002)	0.0239 (0.0418)	−0.011 (−0.034, 0.012)	0.3452 (0.5576)	0.005 (−0.018, 0.028)	0.6536 (0.7224)
Gamma-GT, U/L	0.012 (−0.002, 0.026)	0.0980 (0.1156)	−0.009 (−0.032, 0.013)	0.4173 (0.6260)	−0.021 (−0.044, 0.002)	0.0683 (0.1195)
BUN, mg/dL	0.037 (0.024, 0.050)	<0.0001 * (<0.0001)	0.011 (−0.010, 0.033)	0.3129 (0.5576)	−0.026 (−0.048, −0.004)	0.0187 (0.0436)
Creatinine, mg/dL	−0.016 (−0.026, −0.006)	0.0013 * (0.0025)	0.009 (−0.007, 0.025)	0.2741 (0.5576)	0.025 (0.009, 0.042)	0.0022 * (0.0116)
Uric acid, mg/dL	−0.012 (−0.024, 0.001)	0.0625 (0.0932)	−0.005 (−0.025, 0.015)	0.6174 (0.8103)	0.007 (−0.013, 0.027)	0.5179 (0.6398)

SBP: systolic blood pressure, DBP: diastolic blood pressure, BMI: body mass index, WBC: white Blood Cell, HbA1C: hemoglobin A1C, LDL-C: low-density lipoprotein cholesterol, AST: aspartate aminotransferase, ALT: alanine aminotransferase, Gamma-GT: gamma-glutamyltransferase, BUN: blood urea nitrogen. Using linear regression models to investigate the association between biochemical variables and blood types, adjusted for age, sex, and the first 10 principal components (PCs). *p*-values are shown with BH-FDR adjusted values in parentheses (BH-FDR was calculated across 21 tested traits). Values with *p* < 0.05 are highlighted in orange, and those significant after Bonferroni correction are indicated with *.

**Table 5:** The direction and significance of associations for similar disease categories align between Taiwan Biobank (O vs. non-O) and UK Biobank (genetic correlations from Groot et al., 2020 [4]).

Disease/Trait	Taiwan Biobank (O vs. non-O)	Direction (↑/↓)	Significant (*p* < 0.05)	UK Biobank (Genetic Correlation with Cognition/Education)	Direction (↑/↓ = Risk vs. Higher Cognition)	Significant (FDR < 0.05)	Cross-Population Trend
Coronary Artery Disease (CAD)	No significant association (OR ≈ 1.0)	–	✗	rg = −0.26 (education vs. CAD)	↓	✓	Consistent (↓ risk in higher cognitive function group)
Hyperlipidemia/LDL-C/Cholesterol	OR = 1.16 for A vs. O; ↑ LDL-C (+3.3%)	↑	✓	rg ≈ −0.23 (BMI vs. education; lipid proxy not direct)	↓	✓	Opposite (LDL↑ ↔ lower cognition in UK sample)
Hypertension/Blood Pressure	OR = 0.94 (A vs. O) → lower risk	↓	✓	rg = −0.08 (education vs. systolic BP)	↓	✓	Consistent (↓ in both)
Type 2 Diabetes/HbA1c	HbA1c β = +0.025 (A vs. O) → higher	↑	✓	rg = −0.09 (education vs. T2D)	↓	✗/trend	Opposite (↑ metabolic risk in A type)
Peptic Ulcer Disease	OR = 0.89 (A vs. O), 0.92 (B vs. O)	↓	✓	Not available in UK Biobank panel	–	–	–
Asthma	OR = 1.18 (A vs. O) → higher risk	↑	✓	Not available in UK Biobank panel	–	–	–
BMI/Obesity Index	Slightly higher BMI in non-O (not sig.)	↑	✗	rg = −0.23 (education vs. BMI)	↓	✓	Opposite (↑ BMI in A/B ↔ lower education in UK)

Legend: ↑ = higher risk or value; ↓ = lower risk or value; ✗ = not significant; ✓ = significant.

## Data Availability

The Taiwan Biobank genetic data are subject to controlled access due to privacy policy requirements. Access can be obtained by submitting a formal request to the Taiwan Biobank at biobank@gate.sinica.edu.tw, with responses typically provided within 30 days, subject to approval. Approved users must adhere to data use agreements restricting secondary distribution and non-research use. Source data are provided with this paper.

## Supplementary Materials

The following supporting information can be downloaded at: https://www.mdpi.com/article/10.3390/life15121793/s1, Table S1. Definitions of ABO blood types based on SNPs rs8176746 and rs8176719. Table S2. Definitions of ABO blood types based on SNPs rs8176719, rs635634, and rs7030248. Table S3. The association between clinical conditions and blood type. Table S4. The association between biochemical variables and blood types.

## Abbreviations

The following abbreviations are used in this manuscript:

AMI	Acute myocardial infarction
AST	Aspartate aminotransferase
ALT	Alanine aminotransferase
BMI	Body mass index
BUN	Blood urea nitrogen
CAD	Coronary artery disease
DBP	Diastolic blood pressure
GT	Gamma-glutamyltransferase
HbA1c	Hemoglobin A1C
HDL-C	High-density lipoprotein cholesterol
LDL-C	Low-density lipoprotein cholesterol
MDD	Major depressive disorder
OCD	Obsessive–compulsive disorder
OR	Odds ratio
PCs	Principal components
RBC	Red blood cell
SBP	Systolic blood pressure
SD	Standard deviation
SNP	Single nucleotide polymorphism
sE-selectin	Soluble E-selectin
TG	Triglycerides
VTE	Venous thromboembolism
WBC	White blood cell

## References

[1] Hosoi E. (2008). Biological and clinical aspects of ABO blood group system. J. Med. Investig..

[2] Neshat S., Rezaei A., Farid A., Javanshir S., Dehghan Niri F., Daneii P., Heshmat-Ghahdarijani K., Sotoudehnia Korani S. (2024). Cardiovascular Diseases Risk Predictors: ABO Blood Groups in a Different Role. Cardiol. Rev..

[3] Takagi H., Umemoto T. (2015). Meta-Analysis of Non-O Blood Group as an Independent Risk Factor for Coronary Artery Disease. Am. J. Cardiol..

[4] Groot H.E., Villegas Sierra L.E., Said M.A., Lipsic E., Karper J.C., van der Harst P. (2020). Genetically Determined ABO Blood Group and its Associations With Health and Disease. Arterioscler. Thromb. Vasc. Biol..

[5] Jenkins P.V., O’Donnell J.S. (2006). ABO blood group determines plasma von Willebrand factor levels: A biologic function after all?. Transfusion.

[6] Franchini M., Favaloro E.J., Targher G., Lippi G. (2012). ABO blood group, hypercoagulability, and cardiovascular and cancer risk. Crit. Rev. Clin. Lab. Sci..

[7] Nik A., Mirfeizi Z., Rezaieyazdi Z., Khodashahi M., Danevash S., Sheikh Andalibi M.S., Abbasi M., Sahebari M. (2021). ABO and Rh blood groups in patients with lupus and rheumatoid arthritis. Casp. J. Intern. Med..

[8] Kamil M., Al-Jamal H.A., Yusoff N.M. (2010). Association of ABO blood groups with diabetes mellitus. Libyan J. Med..

[9] Al-Sawat A., Alswat S., Alosaimi R., Alharthi M., Alsuwat M., Alhasani K., Alharthi W. (2022). Relationship Between ABO Blood Group and the Risk of Colorectal Cancer: A Retrospective Multicenter Study. J. Clin. Med. Res..

[10] Chakrani Z., Robinson K., Taye B. (2018). Association Between ABO Blood Groups and Infection: A Meta-Analysis. Sci. Rep..

[11] Obaid J.M.A.S., Al-gashaa F.A.S. (2024). Bacterial Infection versus Viral Infection Preference of ABO Blood Group Phenotype Patients. Jpn. J. Infect. Dis..

[12] Pisk S.V., Vuk T., Ivezic E., Jukic I., Bingulac-Popovic J., Filipcic I. (2019). ABO blood groups and psychiatric disorders: A Croatian study. Blood Transfus.-Italy.

[13] Paul I., Nasrallah H.A. (2023). Association of ABO Blood Types with Psychiatric Disorders: Potential Biomarkers of Genetic Susceptibility?. Biomark. Neuropsychiatry.

[14] Rinieris P.M., Stefanis C.N., Lykouras E.P., Varsou E.K. (1979). Affective disorders and ABO blood types. Acta Psychiatr. Scand..

[15] Garvert L., Baune B.T., Berger K., Boomsma D.I., Breen G., Greinacher A., Hamilton S.P., Levinson D.F., Lewis C.M., Lucae S. (2021). The association between genetically determined ABO blood types and major depressive disorder. Psychiatry Res..

[16] Boyer W.F. (1986). Influence of Abo Blood-Type on Symptomatology among Outpatients—Study and Replication. Neuropsychobiology.

[17] Chester M.A., Olsson M.L. (2001). The ABO blood group gene: A locus of considerable genetic diversity. Transfus. Med. Rev..

[18] Fan C.T., Lin J.C., Lee C.H. (2008). Taiwan Biobank: A project aiming to aid Taiwan’s transition into a biomedical island. Pharmacogenomics.

[19] Chen C.H., Yang J.H., Chiang C.W.K., Hsiung C.N., Wu P.E., Chang L.C., Chu H.W., Chang J., Song I.W., Yang S.L. (2016). Population structure of Han Chinese in the modern Taiwanese population based on 10,000 participants in the Taiwan Biobank project. Hum. Mol. Genet..

[20] Feng Y.A., Chen C.Y., Chen T.T., Kuo P.H., Hsu Y.H., Yang H.L., Chen W.J., Su M.W., Chu H.W., Shen C.Y. (2022). Taiwan Biobank: A rich biomedical research database of the Taiwanese population. Cell Genom..

[21] Wu C.S., Hsu L.Y., Shen C.Y., Chen W.J., Wang S.H. (2025). Validation of Self-reported Medical Condition in the Taiwan Biobank. J. Epidemiol..

[22] Lin W.Y., Wang Y.C., Teng I.H., Liu C., Lou X.Y. (2021). Associations of five obesity metrics with epigenetic age acceleration: Evidence from 2474 Taiwan Biobank participants. Obesity.

[23] Melzer D., Perry J.R., Hernandez D., Corsi A.M., Stevens K., Rafferty I., Lauretani F., Murray A., Gibbs J.R., Paolisso G. (2008). A genome-wide association study identifies protein quantitative trait loci (pQTLs). PLoS Genet..

[24] Wang M., Gao J., Liu J., Zhao X., Lei Y. (2021). Genomic Association vs. Serological Determination of ABO Blood Types in a Chinese Cohort, with Application in Mendelian Randomization. Genes.

[25] Buckwalter J.A., Wohlwend E.B., Colter D.C., Tidrick R.T., Knowler L.A. (1956). Peptic ulceration and ABO blood groups. J. Am. Med. Assoc..

[26] Edgren G., Hjalgrim H., Rostgaard K., Norda R., Wikman A., Melbye M., Nyrén O. (2010). Risk of Gastric Cancer and Peptic Ulcers in Relation to ABO Blood Type: A Cohort Study. Am. J. Epidemiol..

[27] Alkebsi L., Ideno Y., Lee J.S., Suzuki S., Nakajima-Shimada J., Ohnishi H., Sato Y., Hayashi K. (2018). Gastroduodenal Ulcers and ABO Blood Group: The Japan Nurses’ Health Study (JNHS). J. Epidemiol..

[28] Teshome Y., Mekonen W., Birhanu Y., Sisay T. (2019). The association between ABO blood group distribution and peptic ulcer disease: A cross-sectional study from Ethiopia. J. Blood Med..

[29] Hein H.O., Suadicani P., Gyntelberg F. (1997). Genetic markers for peptic ulcer—A study of 3387 men aged 54 to 74 years: The Copenhagen male study. Scand. J. Gastroenterol..

[30] Alkout A.M., Blackwell C.C., Weir D.M. (2000). Increased inflammatory responses of persons of blood group O to *Helicobacter pylori*. J. Infect. Dis..

[31] MacDonald C.J., Madika A.-L., Severi G., Fournier A., Boutron-Ruault M.-C. (2021). Associations between smoking and blood-group, and the risk of dyslipidaemia amongst French women. Sci. Rep..

[32] El-Sayed M.-I.K., Amin H.-K. (2015). ABO blood groups in correlation with hyperlipidemia, diabetes mellitus type II, and essential hypertension. Asian J. Pharm. Clin. Res..

[33] Gong P., Li S., Hu L., Luo S., Li J., Jiang H. (2015). Total cholesterol mediates the effect of ABO blood group on coronary heart disease. Zhonghua Xin Xue Guan Bing Za Zhi.

[34] Gillum R.F. (1991). Blood-Groups, Serum-Cholesterol, Serum Uric-Acid, Blood-Pressure, and Obesity in Adolescents. J. Natl. Med. Assoc..

[35] Li S., Schooling C.M. (2020). A phenome-wide association study of ABO blood groups. BMC Med..

[36] Teng M.S., Hsu L.A., Wu S., Tzeng I.S., Chou H.H., Ko Y.L. (2021). Genome-wide association study revealed novel candidate gene loci associated with soluble E-selectin levels in a Taiwanese population. Atherosclerosis.

[37] Abe Y., El-Masri B., Kimball K.T., Pownall H., Reilly C.F., Osmundsen K., Smith C.W., Ballantyne C.M. (1998). Soluble cell adhesion molecules in hypertriglyceridemia and potential significance on monocyte adhesion. Arterioscl. Throm. Vas..

[38] Li X.Y., Zhang E.H., Zhang P., Liu Y.J. (2012). Relationship between Serum Soluble Cellular Adhesion Molecules Concentration and Cholesterol and Lipoproteins in Patients Referred to Atherosclerosis. Heart.

[39] Aali H., Soofi D., Forghani S.A., Rashki R., Forghani S.N. (2023). The Relationship of Chronic Pulmonary Diseases and Asthma with Blood Groups in Hospitalized Patients. Int. J. Basic Sci. Med..

[40] Alo M.N., Eze U.A., Yaro S.A., Jubril B., Nwanoke N.N. (2015). Relationship between ABO and rhesus blood groups and susceptibility to asthma within Sokoto metropolis, Nigeria. Int. J. Immunol..

[41] Lal T., Sadhasivam M., AK S.P., SJ A.A., Padmavathi R., Khaleeluddin K.B. (2023). An observational study to analyse the association of the ABO and Rh blood group systems with bronchial asthma. Cureus.

[42] Biswas J., Islam M.A., Rudra S., Haque M.A., Bhuiyan Z.R., Husain M., Mamun A.A. (2008). Relationship between blood groups and coronary artery disease. Mymensingh Med. J..

[43] Kutlay Ö., Yalım Z., Keskin-Aktan A., Koca T. (2022). Association between carotid artery disease and ABO blood group. J. Clin. Pract. Res..

[44] He M., Wolpin B., Rexrode K., Manson J.E., Rimm E., Hu F.B., Qi L. (2012). ABO blood group and risk of coronary heart disease in two prospective cohort studies. Arterioscler. Thromb. Vasc. Biol..

[45] Chen Z., Yang S.H., Xu H., Li J.J. (2016). ABO blood group system and the coronary artery disease: An updated systematic review and meta-analysis. Sci. Rep..

[46] Jung E., Kong S.Y., Ro Y.S., Ryu H.H., Do Shin S. (2022). Serum Cholesterol Levels and Risk of Cardiovascular Death: A Systematic Review and a Dose-Response Meta-Analysis of Prospective Cohort Studies. Int. J. Environ. Res. Public Health.

[47] Iselin B.M., Willimann P., Seifert B., Casutt M., Bombeli T., Zalunardo M., Pasch T., Spahn D. (2001). Isolated reduction of haematocrit does not compromise in vitro blood coagulation. Br. J. Anaesth..

[48] Bhandari A., Wagner T. (2006). Self-reported utilization of health care services: Improving measurement and accuracy. Med. Care Res. Rev..

