# Supplementary material for: ABO Blood Type Associations with Physical, Mental, and Biochemical Characteristics in a Taiwanese Population
Source: Life (Basel). 2025 Nov 21;15(12):1793. doi: 10.3390/life15121793 (PMC12733855; doi:10.3390/life15121793)
Supplement: Supplementary file 1 [file life-15-01793-s001.zip › Supplemental Table S1 and S2.pdf]

Supplemental Table S1 Definitions of ABO blood types based on SNPs rs8176746 and rs8176719.

rs8176746	rs8176719	ABO blood types
G/G	T/T	O
T/G	T/T	O
G/G	TC/T	A
G/G	TC/TC	A
T/T	TC/TC	B
T/G	TC/T	B
T/G	TC/TC	AB

Supplemental Table S2 Definitions of ABO blood types based on SNPs rs8176719, rs635634, and rs7030248.

rs8176719	rs635634	rs7030248	ABO blood types
T/T	C/C	G/G	O
T/T	C/C	A/G	O
T/T	C/C	A/A	O
T/T	T/C	G/G	O
T/T	T/C	A/G	O
T/T	T/C	A/A	O
T/T	T/T	A/A	O
T/T	T/T	G/G	A
T/T	T/T	A/G	A
TC/T	T/C	G/G	A
TC/T	T/C	A/G	A
TC/T	T/C	A/A	A
TC/T	T/T	G/G	A
TC/T	T/T	A/G	A
TC/T	T/T	A/A	A
TC/TC	T/T	G/G	A
TC/TC	T/T	A/G	A
TC/TC	T/T	A/A	A
TC/T	C/C	G/G	B
TC/T	C/C	A/G	B
TC/T	C/C	A/A	B
TC/TC	C/C	A/G	B
TC/TC	C/C	A/A	B
TC/TC	C/C	G/G	AB

rs8176719	rs635634	rs7030248	ABO blood types
TC/TC	T/C	G/G	AB
TC/TC	T/C	A/G	AB
TC/TC	T/C	A/A	AB
